# Broadening the Scope of Peer-Mediated Intervention for Individuals with Autism Spectrum Disorders

**DOI:** 10.1007/s10803-017-3429-1

**Published:** 2017-12-08

**Authors:** Mateusz Płatos, Kinga Wojaczek

**Affiliations:** Faculty of Psychology, University of Warsaw, Stawki Str. 5/7, 00-183 Warsaw, Poland

**Keywords:** Autism spectrum disorders, Peer-mediated intervention, Befriending, Adolescent, Adult, Friendship

## Abstract

Peer-mediated intervention (PMI) is most commonly defined as a treatment approach that engages typically developing peers to teach children with autism spectrum disorders (ASD) social skills and increase their social interactions, mainly in a school setting. In this letter, we address the limitations of such understanding of PMI and review the arguments for broadening its scope. In particular, we argue that there is a critical need for research on PMI that focuses on friendship, social participation, and well-being of adolescents and adults with ASD, as well as engages peers in the community settings. In conclusion, we provide a description of a befriending scheme for individuals with ASD to inspire future research and guidelines on PMI.

Peer-mediated intervention (PMI) is a set of practices in which typically developing peers are selected, trained, and supervised to teach or support individuals with autism spectrum disorders (ASD). With over 60 experimental studies published to date (Chan et al. 2009; Chang and Locke 2016; Watkins et al. 2015), PMI is considered one of the most supported and recommended evidence-based practices (EBPs) for children with ASD (Reichow and Volkmar 2010; Wang et al. 2011; Wong et al. 2015). Yet, in this letter we would like to draw attention to significant gaps in research on PMI and propose three extensions of its scope to include: (a) out-of-school interventions that broaden social environments in which PMI can be provided, (b) adolescents and adults with ASD as a target group of PMI, and (c) befriending schemes involving procedures and outcomes related to friendship, social participation, and well-being. As we argue, those additions to PMI are well-matched with current findings on limitations of available treatments and unmet needs of individuals with ASD. These propositions are exemplified by a description of a befriending scheme for adolescents and adults with ASD—*Peer Volunteers ‘Mary and Max’—*that has been developed in Poland.

## Extensions of Peer-Mediated Intervention

Since the first studies on PMI in the late 1970s (Strain et al. 1979), schools and preschools have been a natural setting to provide PMI, ranging from settings in general education classes (Katz and Girolametto 2013) to schoolyards (Kasari et al. 2012) and secluded school facilities (Ganz et al. 2012). Given that school is a primary context for children to socialize with peers, schools seem a good place to implement PMI. The question remains, however, if effects of PMI generalize also to different settings, namely, out-of-school environments, such as extracurricular activities, playground, or hanging out with peers.

Considering difficulties of individuals with ASD in adapting to novel and unstructured situations (Hauck et al. 1995), narrow range of interests (APA 2013) and deficits in executive functions (Rosenthal et al. 2013), informal social and leisure activities may be particularly hard for them to participate. Research suggests that out-of-school participation of children with ASD is limited in a range of activities, social partners, and locations, compared to typically developing children, but differences are more pronounced in informal activities than formal ones (Hilton et al. 2008). A study by Shattuck et al. (2011) showed that about half of adolescents with ASD never see friends after school, are never called by friends, or invited to social activities, and the rates of social participation in their case are substantially lower than in other disability groups. Hence, PMI should encompass out-of-school activities, both organized and unorganized that would provide individuals with ASD environment-specific skills and experiences (e.g. going to the cinema with friends) that cannot be replicated in the school environment.

The second proposed extension of research on PMI concerns the inclusion of adolescents and adults with ASD as its target groups. Out of 42 studies on PMI reviewed by Chan et al. (2009) none referred to participants older than 13 years and only a few more recent case studies (Hughes et al. 2013; Ness 2013) bridge that gap. That coincides with the general scarcity of EBPs for adolescents and adults with ASD (Reichow and Volkmar 2010; Shattuck et al. 2012). However, there are many areas of adolescent and adult functioning in which specifically PMI could provide significant support. From early adolescence the role of friendship grows as a platform for the development of interpersonal competence and intimacy (Buhrmester 1990) and a preparation for romantic relationships (Connolly et al. 2000). It is not surprising that, due to their social difficulties, adolescents with ASD struggle to achieve high social status in a peer group (Symes and Humphrey 2010) and maintain friendships (Orsmond et al. 2004), which results in loneliness (Bauminger et al. 2003), sense of exclusion (Wainscot 2008), and high rates of bullying (Kloosterman et al. 2013), as well as high prevalence of anxiety, depression (Strang et al. 2012), and suicidal ideation (Mayes et al. 2013) in that group. Applying PMI to foster peer relations and friendship in adolescents with ASD could be a preventive measure against many of these negative outcomes (Bradley 2016).

The third and last proposed extension of research on PMI concerns its core procedures, aims, and intended outcomes. We argue that current focus of PMI for individuals with ASD on teaching social skills limits the potential of peer involvement. Teaching new skills is considered only one type of relationship-based voluntary schemes, the type usually referred to as ‘mentoring’. The other end of the continuum of that broad category of schemes is represented by ‘befriending’ programs, that are focused on building supportive, friendship-like relationships, involving participation in joint pleasurable activities and conversations about common interests, but also some level of disclosure and emotional support (Thompson et al. 2016). Such a definition does not imply that the transfer of skills is not a goal and does not occur in befriending programs, but that transfer is embedded in a long-term, reciprocal relationship. Moreover, a different kind of skills is taught in befriending relationships, namely, friendship skills. The recognition that friendship requires some specific social skills is reflected in the emergence of evidence-based friendship trainings (Laugeson and Frankel 2010). However, we argue that learning friendship skills must involve the experience of at least some qualities of friendship—such as shared enjoyment, emotional bond, equality, and mutuality—in the context of a long-term relationship. For example, it is hard to learn how to manage jealousy of a friend without experiencing that feeling in a real-life situation. Therefore, we propose that befriending relationship can be a basis for socially and ecologically valid intervention that would teach individuals with ASD friendship skills generalizable to non-facilitated friendships.

While befriending schemes can serve as viable means of teaching individuals with ASD friendship skills, this should not be regarded as the only, and perhaps, the most important aim of such intervention. Befriending programs are widespread in mental health services, aimed at reducing loneliness, isolation, and affective symptoms in vulnerable populations (Thompson et al. 2016). As noted in a comprehensive review of EBPs by Wong et al. (2015), interventions for individuals with ASD rarely target their emotional well-being. We propose that providing a person with ASD with a stable, supportive, and meaningful relationship with a peer can attenuate some negative emotional outcomes linked to social exclusion of adolescents and adults with ASD, as well as increase their level of self-competence and social participation.

Lastly, research suggests not only a need of many individuals with ASD to have friends (Eaves and Ho 2008; Mazurek 2014; O’Hagan and Hebron 2017) but also a clear need for befriending services, as can be illustrated with data from three countries. A large-scale survey undertaken in England by the National Autistic Society (Rosenblatt 2008) showed that 33% of adults with ASD (N = 1.179) would like to participate in a befriending scheme, while only 5% have it provided. In an Australian survey (Autism Spectrum Australia 2012) of high-functioning adults with ASD, about 35% of respondents (N = 313) declared an unmet need for a mentor or befriender, while 28% already had access to mentoring or befriending support. Finally, a survey of adolescents and adults with ASD in Poland (Płatos et al. 2016), indicated that 43% of respondents (N = 117) would like to participate in a befriending scheme. Moreover, 49% of parents in the English sample and 77% of parents in the Polish sample believed that their adolescent or adult child would benefit from befriending. Together, these results indicate that befriending services are greatly needed by individuals with ASD.

## Peer Volunteers ‘Mary and Max’

The above propositions of extending the scope of PMI will be exemplified by the description of Peer Volunteers ‘Mary and Max’—PMI that has been developed in Poland since 2012. Peer Volunteers ‘Mary and Max’ is a manual-based befriending scheme for high-functioning adolescents (from 12 years old) and adults with ASD (that we refer to as ‘participants’). The focus of the program is to facilitate the one-to-one relationship between a participant and a volunteer, based on their common interests and joint leisure activities. The aim of arranging such a relationship is to provide a person with ASD a positive, supportive experience associated with a peer, enhance his or her self-perceived interpersonal competence, and increase social participation, thereby decreasing the sense of loneliness and isolation.

The key element of the program is the process of recruitment, selection, and matching of participants and volunteers. This process aims at (a) finding individuals with ASD who have intrinsic motivation to have a friend and spent time together, (b) finding peers who present high motivation to volunteer, good social skills, and emotional maturity, (c) matching participants and volunteers into pairs on the basis of their common interests, preferred activities, age, and area of living. The successful matching is the precondition for a safe, self-motivated, and enjoyable relationship the program is to facilitate. Before the program starts, volunteers undergo a 2-day training based on experiential learning that helps them understand the needs of people with ASD and their role as volunteers.

The core of the program consists in one-to-one, weekly get-togethers of a participant and a volunteer that involve various shared activities, planned and chosen by both sides. Those get-togethers are unsupervised either by professionals or parents and are held mainly in the public (e.g. in a park, a cinema, or a café), enhancing participants’ access to the community life. Both participants and volunteers receive professional support in maintaining the relationship, provided by a psychologist who is assigned to the dyad throughout the program. The psychologist holds monthly individual consultations with the participant and the volunteer, working on their initiative, motivation, planning and organizing of get-togethers, expressing emotions, and essentially on the mentalizing skills. Eventually, the psychologist helps participants and volunteers decide whether they want to continue their relationship outside the scheme or resolve it in a gradual and supported process. Although evolving a befriending relationship into a friendship is not the aim of the scheme, most participants and volunteers express their willingness to do it, thus the psychologist’s role is to help them make such a transition and take full responsibility for the relationship.

## Conclusions

In this short article we argued that the scope of PMI should not be limited to teaching children with ASD social skills in a school setting. Although this type of PMI should certainly be continued and developed, we presented evidence that it would be beneficial to include peers in the support of adolescents and adults with ASD in the community and using befriending schemes to enhance their social participation and well-being. Importantly, the need for such services is clearly expressed by individuals with ASD themselves. Where those services have already existed, they should be backed by empirical research to inform international guidelines and EBP for PMI.
